# Novel roles for JNK1 in metabolism

**DOI:** 10.18632/aging.100192

**Published:** 2010-08-31

**Authors:** Bengt F. Belgardt, Jan Mauer, Jens C. Brüning

**Affiliations:** ^1^ Institute for Genetics, Department of Mouse Genetics and Metabolism, Center for Molecular Medicine, University of Cologne (CMMC), Cologne Excellence Cluster on Cellular Stress Responses in Aging-Associated Diseases (CECAD), 2^nd^ Department for Internal Medicine University of Cologne 50674 Cologne, Germany; ^2^ Max Planck Institute for the Biology of Ageing, 50674 Cologne, Germany

**Keywords:** JNK1, obesity, insulin resistance, CNS

## Abstract

Activation of stress-kinase signaling has recently been recognized as an important pathophysiological mechanism in the development of diet-induced obesity, type 2 diabetes mellitus and other aging-related pathologies. Here, c-Jun N-terminal Kinase (JNK) 1 knockout mice have been shown to exhibit protection from diet-induced obesity, glucose intolerance, and insulin resistance. Nonetheless, the tissue-specific role of JNK1-activation in the development of the metabolic syndrome has been poorly defined so far. Recently, it was demonstrated that JNK1 signaling plays a crucial role in the central nervous system (CNS) and in the pituitary to control systemic glucose and lipid metabolism partially through regulation of hormones involved in growth and energy expenditure.

## Insulin signalling and its negative regulators in aging-associated diseases

The insulin/insulin-like signalling pathway is highly conserved throughout the animal kingdom. Whereas its predominant role in mammals is the control of metabolic homeostasis and its deregulation leads to the development of diabetes mellitus, lowering insulin/insulin-like signalling in *c. elegans, d. melanogaster* and *m. musculus* has been implicated in lifespan extension [1-5].

**Figure 1.**
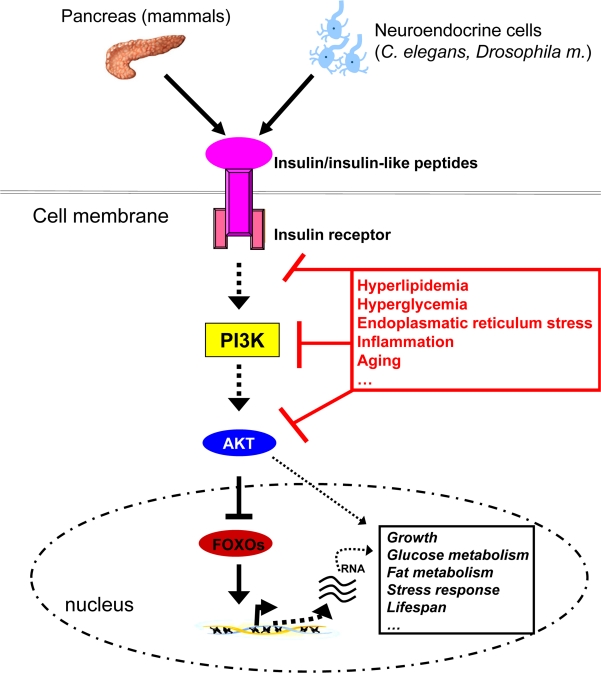
Insulin-like signalling plays a central role in growth, metabolism and the aging process. Insulin, derived from pancreatic beta-cells in mammals or insulin-like peptides derived from neuroendocrine cells in invertebrates signals via binding to and activation of the membrane bound receptors. This event subsequently activates PI3K, which through phosphorylation of membrane lipids (phosphorinositides) regulates activity of the downstream kinase AKT. AKT eventually phosphorylates forkhead transcript-tion factors such as FOXO1, which are then exported from the nucleus and degraded. FOXOs regulate transcription of many genes involved in glucose and lipid metabolism, growth, stress response and the aging process. Thus, insulin-like signalling is able to control all of these processes through FOXO regulation and other signalling cascades, in the end impinging on crucial physiological processes and lifespan itself. Nonetheless, chronic intake of energy-dense food coupled with little physical activity leads to hyperlipidemia and hyperglycemia, which through several mechanisms (including JNK1 activation) reduce cellular insulin sensitivity, thereby disrupting metabolic homeostasis.

The anabolic peptide hormone insulin is secreted from the pancreas in response to an increase of blood glucose concentrations. It acts on the liver to reduce hepatic glucose output and it promotes glucose and lipid uptake into peripheral tissues such as adipose tissue and skeletal muscle. Binding of insulin or insulin-like peptides to their receptor leads to recruitment of insulin receptor substrate (IRS) proteins, subsequently activating two major signalling branches: the phosphatidylinositol 3 kinase (PI3K)-pathway and the mitogen-activated protein kinase (MAPK)-pathway [6,7]. PI3K activity mediates activation of the kinase AKT, which phosphorylates and thereby deactivates forkhead transcription factors (FOXOs). FOXOs are transcriptional regulators of genes involved in metabolism and growth [8]. Activation of the PI3K/AKT/FOXO axis mediates many of insulin's and insulin-like peptides’ effects, including e.g. regulation of growth, glucose/fat metabolism, stress response and lifespan (Figure 1) [9]. Besides the expression and activation of this pathway in peripheral organs, the insulin/insulin-like signalling machinery is also expressed and active in the central nervous system (CNS) where it regulates fertility and body weight [10-12]. Furthermore, it was recently demonstrated that insulin action in the CNS also controls peripheral glucose and fat metabolism [13-15].

In the last decade, several studies have demonstrated that central as well as peripheral insulin signalling can be drastically impaired by a variety of obesity- and/or aging-associated parameters such as hyperlipidemia, hyperglycemia, endoplasmatic reticulum (ER) stress and inflammation [16-19]. Following this, the incidence of numerous aging-associated diseases such as diabetes mellitus and obesity has created an urgent necessity to define the mechanisms underlying energy intake and expenditure, and to identify molecular targets for pharmacological intervention.

## JNK1 and aging-associated diseases

In 2002, the group of Gökhan Hotamisligil revealed that mice deficient for the stress mediator c-Jun N-terminal Kinase (JNK) 1 are protected from the development of high fat diet-induced obesity and glucose intolerance, as well as insulin resistance [20]. Nonetheless, it remained unclear, in which tissue(s) JNK1 might act to impair energy and glucose homeostasis under conditions of diet-induced obesity.

The family of JNK kinases can not only be activated by cytokines, but also by endoplasmatic reticulum (ER) stress and hyperlipidemia, all of which are elevated in obesity and/or diabetes mellitus [21]. Previous data indicated that upon activation, JNK1 mediates inhibitory serine phosphorylation of IRS proteins, thereby impairing insulin action [22]. Interestingly, it was recently reported that mutation of the most frequently investigated JNK1 phosphorylation site, Ser307, augments (and not blocks) insulin resistance in obese mice, possibly pointing to either adaptive mechanisms during development or additional parallel pathways by which JNK1 can affect metabolism [23].

## JNK1 and CNS insulin sensitivity

In the last year, JNK1 has been conditionally inactivated in several peripheral classically insulin-sensitive tissues including adipose tissue, muscle and liver [24-26] (Figure 1). Nevertheless, none of these mouse models fully recapitulated the protection from obesity and diabetes observed in conventional knockout mice opening the possibility that JNK1 activation also in the CNS may contribute to its effects on energy and glucose metabolism.

JNK activation in the hypothalamus during obesity development has been linked to endoplasmatic reticulum stress, inflammation, or hyperlipidemia [17, 27-29]. Notably, during our studies of lipid-induced hypothalamic leptin resistance, we observed that intra-cerebroventricular (icv.) injection of saturated fatty acids such as palmitate, induced activation of hypothalamic IKK, whereas activation of JNKs was not readily detectable *in vivo*[30]. To firmly address the question if JNK1 activation in the CNS will give rise to dysfunctional energy homeostasis, mice lacking JNK1 in all neurons (called JNK1^∆Nes^) were generated by crossing mice with a loxP-flanked JNK1 allele with those harbouring a Nestin-Cre gene, which is generally used to ablate a gene of interest in neurons and astrocytes in the CNS [10,31]. In line with previous studies, JNK1 ablation in the CNS did not affect leptin sensitivity, independent of route of administration (intraperitoneal or icv.). Thus, it was asked if JNK1, in line with its putative role in regulating peripheral insulin sensitivity, would also affect insulin signalling in the CNS, which is crucial for energy homeostasis [10,13,14]. JNK1^∆Nes^ mice were highly sensitive to the anorectic effect of centrally applied insulin, even when given at doses that had no effect on control mice [31]. In line with the notion that insulin affects body weight and glucose homeostasis mainly by its action in the hypothalamus [32], we demonstrated that high fat diet-fed JNK1^∆Nes^mice remained insulin sensitive in the hypothalamus [31]. This has also been independently demonstrated recently in conventional JNK1 knockout mice [33]. These data indicate that JNK1 ablation in the CNS retains hypothalamic insulin signalling under conditions of positive energy balance. Nonetheless, it is not clear if this effect is solely derived from lack of JNK1 in hypothalamic neurons, or indirectly mediated by other, JNK1-deficient extra-hypothalamic neuron populations with synaptic connections onto hypothalamic neurons. Thus, generation of mice with JNK1 deficiency in specific hypothalamic neuron populations will help to understand the cell-type specific role(s) of JNK1 in the hypothalamus.

**Figure 2.**
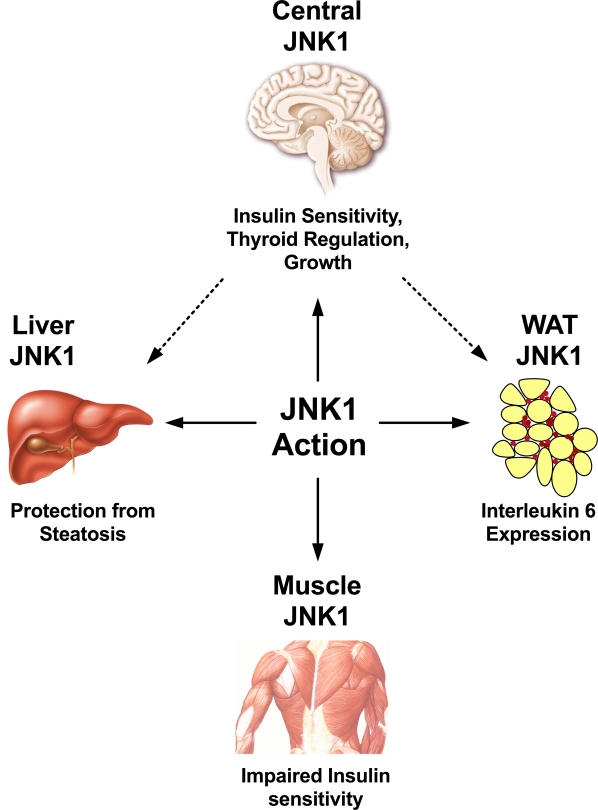
JNK1 represents a crucial regulator for a wide spectrum of physiological processes. In the white adipose tissue, JNK1 has been demonstrated to regulate expression of interleukin 6, which upon release into the circulation may act on the liver to decrease hepatic insulin sensitivity. Hepatic JNK1 action may downregulate insulin degradation, thus improving insulin half-life, and protecting from steatosis. JNK1 action in the skeletal muscle does impair local insulin sensitivity, although systemic glucose homeostasis is mostly unaffected. In the central nervous system, JNK1 is a negative regulator of insulin sensitivity, the thyroid axis and growth, although the exact neuron populations mediating these effects have not been defined yet.

## JNK1, growth and energy expenditure

During metabolic phenotyping, we noticed increased energy expenditure in JNK1^∆Nes^ mice, even when corrected for lean body mass [31]. Accordingly, we found increased circulating levels of the thyroid hormone thriiodothyronine (T3), in the presence of elevated concentrations of its releasing hormone, namely thyroid stimulating hormone (TSH), a finding which was independently reported by Roger Davis and colleagues [31,34]. However, hypothalamic expression of thyroid releasing hormone (TRH), which itself represents the upstream regulator for expression and release of TSH in the pituitary, was unchanged [31].

While Nestin-Cre mice have been widely used for pan-neuronal (and astrocyte-specific) ablation of genes, Nestin is also expressed in a stem cell population in the pituitary [35]. Thus, the deleted JNK1 allele was also detected in pituitaries of JNK1^∆Nes^ mice, indicating that the change in TSH expression and subsequent T3 may be due to a pituitary-autonomous effect [31]. Along this line, the expression of the receptor for TRH, TRHR, was increased by JNK inhibition in pituitary cells *in vitro*, akin to the increased expression of TRHR in pituitaries of JNK1^∆Nes^ mice*in vivo* [31].

Although energy expenditure was increased in JNK1^∆Nes^ mice, body fat mass was not changed compared to controls, indicating that JNK1^∆Nes^mice were not protected from obesity itself, at least during the first four months of age [31]. On the other hand, JNK1^∆Nes^ mice demonstrated reduced body weight either on normal chow diet (ND) or on HFD. Thus, it was asked whether reduced somatic growth may account for the reduced body weight. Indeed, activity of the growth hormone (GH) - insulin-like growth factor (IGF) 1 axis, which controls somatic growth, was reduced [31,36].

## Does JNK1 inhibition mimic caloric restriction?

When exposed to HFD, JNK1^∆Nes^ mice not only demonstrated protection against systemic glucose intolerance and insulin resistance, but also showed reduced hepatic steatosis, and importantly, an anti-inflammatory gene expression pattern in the adipose tissue [31].

So far, a major intervention known to prolong life (and protect against the plethora of aging-associated diseases) is caloric restriction (CR). Strikingly, CR itself reduces circulating levels of GH in rodents, and inhibition of this decrease may negate the beneficial effects of CR, while mice with mutations in this pathway show longer life span as well as protection against systemic insulin resistance [37-40]. Notably, it is only poorly understood, how CR regulates the GH-IGF1 axis on a molecular level. Upon HFD, JNK activity is increased both in the hypothalamus, but strikingly also in the pituitary of mice, indicating that JNK1 might directly regulate the GH-IGF1 axis in these tissues. In addition, it has been shown that overfeeding increases somatic growth, and HFD increases naso-anal length, in accordance with increased expression of growth hormone releasing hormone receptor (GHRHR) in the pituitary [31]. Thus, we speculate that JNK1 might act as a sensor for nutrients, and thus regulate both energy expenditure and growth in accordance with energy levels.

JNK activation upon obesity may also be interpreted as a stress-resolving response and have beneficial effects under specific circumstances. Thus, JNK-mediated regulation of forkhead transcription factors offers protection from cellular stress, at least in invertebrates [41,42]. Furthermore, signal duration, strength and spatio-temporal distribution may play a role for the net outcome of JNK activation. Eventually, it seems possible that JNK1 regulates either growth or thyroid axis, indirectly affecting one another. Further analysis of cell type-specific JNK1 knockout mice will help to define the roles of this stress kinase in the pathophysiology of obesity, diabetes mellitus and other aging-related diseases.
